# Case Report: Feasibility of a Novel Virtual Reality-Based Intervention for Patients With Schizophrenia

**DOI:** 10.3389/fpsyg.2021.642590

**Published:** 2021-02-26

**Authors:** Edit Vass, Viktória Simon, Zita Fekete, Balázs Kis, Lajos Simon

**Affiliations:** ^1^Department of Psychiatry and Psychotherapy, Faculty of Health, Semmelweis University, Budapest, Hungary; ^2^Institute of Behavioral Sciences, University of Debrecen, Debrecen, Hungary; ^3^Department of Psychiatry, Szabolcs-Szatmar-Bereg County Hospital and University Teaching Hospital, Nyíregyháza, Hungary

**Keywords:** virtual reality, case report, theory of mind, schizophrenia, simulation

## Abstract

Schizophrenia is a severe and disabling mental illness, associated with persistent difficulties in social functioning. While gaining and retaining a job or staying socially integrated can be very difficult for the patients, the treatment of poor functionality remains challenging with limited options in pharmacotherapy. To address the limitations of medical treatment, several interesting and innovative approaches have been introduced in the field of psychotherapy. Recent approaches incorporate modern technology as well, such as virtual reality. A potential therapeutic benefit of virtual reality is particularly significant when an interpersonal dimension of the problem needs to be addressed. One example is a Virtual Reality based Theory of Mind Intervention (VR-ToMIS), a novel method, which enables patients to practice complex social interactions without the burden of real-life consequences. Our paper presents a case report showing promising results of VR-ToMIS. Ms. Smith is a 50- year-old patient who has been suffering from schizophrenia for 20 years. Although in her case there was no problem with compliance throughout the years, she had severe problems regarding social functionality. With VR-ToMIS, she improved in ToM and communicative-pragmatic skills. The effects of the intervention went beyond the increased scores of the tests. Before the intervention there was a risk of the patient becoming unemployed as she was unable to follow the main principles of communicative exchange. Usually, her contribution was more informative than was required. After the intervention her communication became more balanced and she could retain her job. This case suggests that VR-ToMIS may be a promising tool for treating social disfunction in schizophrenia.

## Introduction

Schizophrenia is a chronic mental disorder affecting up to 1% of the population (Saha et al., 2005). While medical treatment primarily aims at the elimination of positive and negative symptoms, it has limited effect on concomitant functional deficits (Leifker et al., 2009; Kovács, 2011). To address this limitation several novel and innovative approaches have been introduced in the field of psychotherapy. During this process, social cognitive interventions have also recently become popular (Kurtz and Richardson, 2012; Vass et al., 2018).

Published findings on the functional significance of social cognitive deficits provide a stable theoretical basis for new developments (Penn et al., 1997; Mancuso et al., 2011; Green et al., 2015). The role of one of the subcomponents, the Theory of Mind (ToM), is considered to be more directly related with functional outcomes (e.g., employment) than other fields of cognition, which highlights ToM deficit as a potential treatment target (Brüne, 2006; Bell et al., 2009; Bora et al., 2009).

Despite early attempts at improving ToM skills in schizophrenia leading to promising results, these methods have often been criticized for not being able to fully cover the complexity (both cognitive and affective aspects) of ToM(Kurtz and Richardson, 2012; Vass et al., 2018). Typically, these interventions use the technique of analyzing social interactions observed in short film clips or displayed on comic strips or by using story vignettes (Kurtz et al., 2016; Tan et al., 2016). Such training materials may not be suitable for grasping the interpersonal dimension of ToM. However, incorporating modern technology, especially immersive virtual reality (VR) might be a promising option in resolving the problem (Vass et al., 2018). VR technology enables the simulation of social interactions where patients have the possibility to practice social skills without the burden of real-life consequences. One of the recently developed interventions that combines cognitive and behavioral therapy techniques and VR technology aims to take advantage of the potential of immersive simulation. VR-ToMIS (=**VR** based **ToM I**ntervention in **S**chizophrenia) is a VR based targeted ToM intervention, especially designed for stable outpatients with schizophrenia. VR-ToMIS primarily uses virtual roleplays to help patients improve their social skills (Vass et al., 2018, 2019). During its development, an iterative testing process was used, based on the published guidelines of the UK Medical Research Council (MRC) on the development and evaluation of multi-component interventions (Craig et al., 2013). The process has proved quite promising and pilot results on its feasibility have already been published. VR-ToMIS improved several aspects of ToM ($\eta_{\text{p}}^{2}$ = 0.24–0.46) and pragmatic skills ($\eta_{\text{p}}^{2}$ = 0.22–0.39), while negative symptoms ($\eta_{\text{p}}^{2}$ = 0.58) were significantly reduced when compared to control conditions. Self-reported Quality-of-Life scores did not change significantly. Yet, close relatives of the patients reported observable and obvious changes in the patients' attitudes and behavior during social interactions. Furthermore, patients found the intervention interesting and tolerated it well. Although the mentioned results show that VR-ToMIS might be beneficial for schizophrenic patients, further study on a larger sample is needed to confirm its short-term and possible long-term effects (Vass et al., 2020).

Here, we present a case report of a chronic schizophrenia patient, showing the advantages of this novel intervention. Ms. Smith has been suffering from schizophrenia for 20 years. Although she responded well to antipsychotic medication, she still suffers from severe functional deficits. She has become socially isolated throughout the years, and before the VR-ToMIS intervention she also had difficulties in retaining her job.

## Case Presentation

### History of the Patient

Ms. Smith, a divorced Caucasian female in her fifties, has been suffering from schizophrenia for the last 20 years. Although most of her symptoms have been alleviated, she still has severe functional difficulties.

She experienced her first symptoms in 2000, after having severe familial conflicts. She started to think that even her family had been conspiring against her, which caused her to make reports against her family members to the police. She experienced thought-reading and showed disorganized behavior. She was hospitalized and first diagnosed with “Schizophreniform disorder,” based on the diagnostic criteria of DSM-IV in 2000, and Haloperidol was started (electronic database was only introduced after 2004 in Hungary, therefore, only information obtained from the patient is available for that period) (American Psyhicatric Association, 2000). Although she suffered from unpleasant side-effects, she had good insight and accepted the treatment. In 2001, considering the frequent delusions and behavioral changes, paranoid type schizophrenia was diagnosed.

While her symptoms improved with medication, management of various life situations caused her severe problems and she also noticed a decline in her social skills. She often found it hard to recognize others' needs, intentions or feelings. She considered these difficulties as a life-changing aspect of her illness, which radically altered her life in many ways: Saha et al. (2005). She often had conflicts with her ex-husband and with her colleagues (Leifker et al., 2009). She was on the verge of dismissal several times (Kovács, 2011). Finally, she lost the few friends she had, even her sons turned away from her.

She was hospitalized four times (three times before 2005, and once in 2018), and tried various medications (Table 1). Additionally, she received psychotherapy that helped her understand how her way of thinking differed from others' (Table 1). However, she had difficulties in transferring the learned skills into real life interactions.

**Table 1:** Pharmacological treatments and psychotherapeutic interventions.

**Medication**	**Dosage**	**Treatment duration**	**Comments**
**Pharmacological treatments**
Haloperidol	Unknown	2000–2007	No data is available in electronic database before 2007 According to the patient she was hospitalized three times before 2005. In all three cases, only the dosage was changed. Medication was stopped because of side effects: dizziness, headache, muscle-weakness, tremor
Risperidone	2 × 2 mg/day	2007.07.12–2013.02.07.	Instead of Haloperidol Stopped because of galactorrhea
Aripiprazole	15 mg/day	2013.05.09–2018.08.30.	Instead of Risperidone. Stopped because of anemia and thrombocytopenia.
Quetiapine	50 mg/day	2017.12.14–2018.05.05.	In conjunction with Aripiprazole No details on the reasons of adding. Stopped because of hallucinations, and depressed mood.
Olanzapine	10 mg/day	2018.04.05–2018.04.12.	Instead of Aripiprazole and Quetiapine Dosage reduction because of sedative side effects.
Duloxetine	30 mg/day	2018.04.05–2018.09.28.	Started in conjunction with antipsychotic medication because of depressed mood.
Olanzapine	5 mg/day	2018.04.12–2018.06.13.	Reduced dose Stopped because of agitation, disorganized behavior, paranoid delusions and impulsivity. Hospitalization was needed.
Paliperidone	150 mg/4 weeks	2018.06.13–ongoing	Started in replacement of Olanzapine Same dosage throughout the study, no additional medication was needed.
**Psychotherapeutic interventions**		
Psychoeducation	During hospitalization and before any changes in medication		The patient found the information useful, which helped her to accept the diagnosis.
Supportive therapy	1 h/session/ week	for 10 weeks in 2015	The patient found the process too slow.
Problem solving group	2 h/week	for 20 weeks in 2017	It was found useful by the patient in solving specific problems, but the coping skills didn't improve in general, based on her subjective evaluation.
Metacognitive training	1 h/sessions, 2 times/week	for 9 weeks in 2018	The patient liked the training and found it interesting, but she had problems with using the learnt skills in real life situations.

At the time of the baseline visit, the patient was stabilized on Paliperidone. Her last psychotherapy finished 1 year prior. Ms. Smith received antihypertensive medication and had no other condition requiring medical treatment.

## Treatment Methods – Course of the Intervention

Upon the first time we met, Ms. Smith expressed suspiciousness without reaching the severity of delusion. She was unable to follow the main principles of communicative exchange. Usually, her contribution was more informative than was required. She still had her job, but she was on the verge of dismissal due to the above-mentioned problems with communication.

Clinical assessment for symptomatology was administered by a trained psychiatrist (BK) using the Positive and Negative Syndrome Scale (PANSS) (Kay et al., 1987; Shafer and Dazzi, 2019). Assessments for ToM, pragmatic language skills and Quality-of-Life on the other hand was ascertained by a licensed clinical psychologist (ZF), consisted of Repeated Battery for the Assessment of Neuropsychological Status (RBANS), Wisconsin Card Sorting Test (WCST-64), Baron-Cohen Minds in the Eyes Test (BCMET- as main inclusion criteria was <22 points, indicating Theory of Mind deficit), Faux pas test, Cartoon stories task, the Hungarian Metaphor and Irony test, and Lancashire Quality-of-Life Profile (LQoLP) (Baron-Cohen and Knight, 1998; Randolph et al., 1998; van Nieuwenhuizen et al., 2001; Varga et al., 2008; Fernández-Abascal et al., 2013; Heaton et al., 2021). Ms. Smith completed all evaluations at baseline, post-treatment and at 3 months after the end of the intervention.

Informed consent was obtained prior to inclusion, including permission for the publication of this case report (Reference number of the ethical approval of the Local Research Ethics Committee: SE-TUKEB 150/2016).

### VR-Based Intervention

Ms. Smith participated in a 9 week VR-based intervention (1 h/session/week) in an individual setting led by a licensed clinical psychologist (EV) under the supervision of an experienced psychotherapist (LS). We used the protocol of the ongoing efficacy study/previously published feasibility study of VR-ToMIS (Vass et al., 2020). VR-ToMIS applies an immersive VR software (vTime), with a Samsung GearVR head mounted display built in a Samsung S7 smartphone, and a Simple Controller (www.vTime.net).

### Education Session

Education sessions served the purpose of familiarizing Ms. Smith with the used technology. Thus, she had the possibility to explore various virtual destinations and meet avatars provided by vTime. VR environments consisted of realistic simulations of various places (e.g.,: Chinese restaurant, coast, office) and an avatar visible from a first-person perspective. Ms. Smith learned to navigate between the virtual locations, and also had the opportunity to design her own avatar (including hair, body type, skin color, dress). In the second part of the session, she met another avatar. She was requested to express her feelings and impressions on the avatar and the situation, so we could redesign it to make the situation more comfortable for her (even the outfit of the avatar, or distance between the avatars). This step also served as the basis for an initial cognitive intervention with the aim of highlighting the possible effects of environmental factors on the interpretation of both the situation and her behavior. At the end of the session, possible side effects and a sense of presence were assessed by using Simulator Sickness Questionnaire and Presence Questionnaire (Kennedy et al., 1993; Witmer and Singer, 1998).

### VR-Sessions

**Figure 1:**
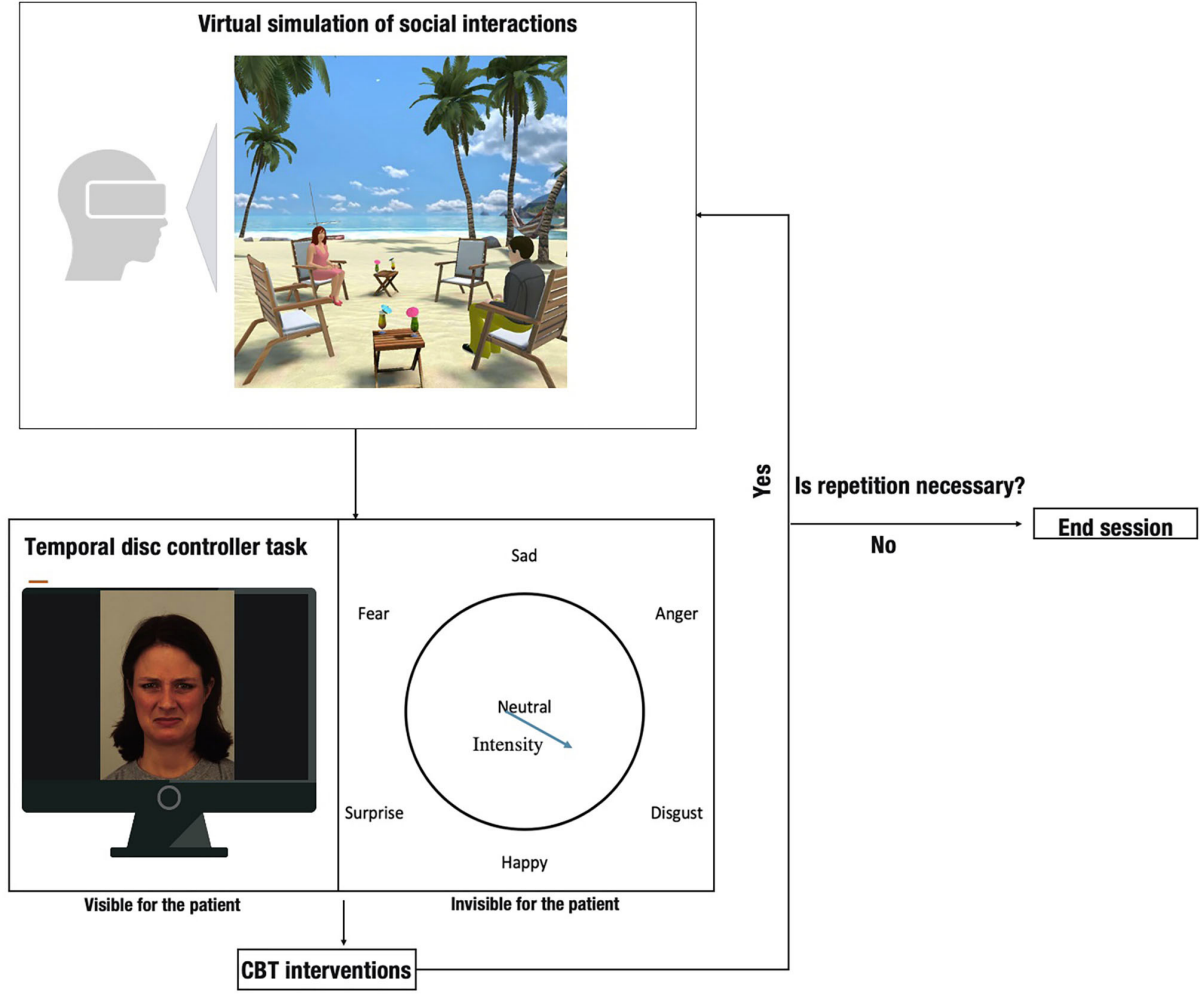
Structure of the intervention (The image illustrating the virtual simulation was used with permission from vTime).

The structure of each session was the same (Figure 1). After a short warm-up, a brief description of the VR-simulation was given to Ms. Smith (e.g.,: session 8: “You are having dinner in a restaurant. A man approaches you”). Then the patient was given the opportunity to ask questions about the situation and design her own avatar appropriate to the situation. The other avatar was designed by the therapist prior to the session.

During the simulation Ms. Smith was asked to participate in a virtual conversation with the avatar in one of the immersive VR destinations of vTime. The dialogs were quite short and aimed to reveal ToM deficits to address in future therapeutic interventions. The virtual conversations were initiated by the avatar controlled by the therapist. All sentences of the dialogues were pre-recorded and played one-by-one following the patient's reaction. To avoid communication dead ends, in the case of some sentences, alternative responses were recorded so that the therapist could choose one which best fits the patient's reaction.

Each simulation was followed by an interactive task using another software, called Temporal Disc Controller (TDC), which is a validated tool to assess emotion perception (Takács and Kiss, 2003; Csukly et al., 2004). The TDC task served as an aid to visualize the inferred emotions of the therapist-controlled avatar (“the other”) by the patient after the simulation. The TDC shows an avatar's 3D face on the computer screen. The emotions on the face can be displayed by moving a cursor on the screen. Right after the TDC task the therapist used cognitive and metacognitive techniques to help Ms. Smith understand how her thoughts and feelings play an influential role in her behavior, and identify maladaptive behavioral patterns applied during the simulation. For instance, when the avatar was bald, it evoked bad memories and made her attitude more sensitive toward him. When circumstances were changed (the avatar had hair) her suspicion became moderated.

After the therapeutic interventions Ms. Smith was encouraged to re-enter the same simulation to “virtually” test the validity of the therapeutic interventions and new behavioral patterns. Minor differences might have occurred between the simulations based on the patient's feedbacks (e.g., the outfit of the other avatar, the distance between the avatars). The described process (simulation-TDC task-CBT intervention – re-simulation) could be repeated up to 3 times per session.

Simulations on different topics were used in each session characterized by different levels of complexity. At the end of each session, individualized homework was given to the patient with the aim of facilitating learning transfer effects. Possible side effects and sense of presence were examined at the end of each session, as outlined above.

## Outcome

### Qualitative Results

#### Impressions – Psychologist

At the beginning Ms. Smith gave extreme reactions within VR simulations. She either didn't respond to the avatar, or her responses were long and more detailed than was required. Environmental factors also had an effect on her attitude during the simulation (e.g., when she found the virtual room too tidy, she thought that she didn't deserve to be there and assumed that the other person thought so too and might have bad thoughts about her. Hence, her interpretations of the situation were based on the thoughts and feelings mentioned above and not on the actual behavior of the other person). As she explained, these reactions were consistent with her real-life behavior. Initially, cognitive and metacognitive techniques proved insufficient to make her realize how her experiences, feelings and thoughts affected her reactions during a particular social interaction. However, when virtual environmental factors were changed and she was encouraged to participate in the same simulation again, she recognized that her feelings or reactions had indeed changed accordingly. As the intervention progressed, virtual behavioral experiments helped the patient to also become more receptive to cognitive techniques.

TDC also had an interesting effect on her. At first, she found it hard to display the inferred feelings on an avatar's 3D face on a computer screen. Sometimes her difficulties with this task caused her to refuse even attempting it. Encouraging Ms. Smith to verbally express and imitate emotions intended to display prior to the actual TDC task helped her slowly become accustomed to it. After a while the presented and verbally described emotions almost completely overlapped.

Despite initial difficulties Ms. Smith enjoyed VR-ToMIS, and even minor successes reinforced her efforts.

At the end of the intervention the communication style of Ms. Smith became more balanced. She adhered more to the main principles of communication described by Grice (Mazza et al., 2008). She was more able to pay attention to the other person's mental state, and was less prone to mistrust.

### Impressions – Ms. Smith and Her Employer

After the intervention, a short post-intervention semi-structured interview was used to evaluate Ms. Smith's thoughts and feelings toward VR-ToMIS. She found VR-ToMIS interesting, enjoyable, realistic and easy to use. She highlighted that VR-ToMIS had an important role in her rehabilitation and that it helped her improve her communication and retain her job. Although she still felt isolated, she also felt more capable of getting involved in meaningful relationships.

Our intention was to also evaluate the changes in Ms. Smith's behavior attributed to the intervention from the perspective of someone close to her. As we couldn't reach any of her close relatives, we visited her employer with Ms. Smith's permission. They have been working together for 19 years and are in daily contact. The employer noticed positive changes in Ms. Smith's willingness to initiate a conversation and in her willingness of being engaged in them. She also noticed that Ms. Smith was able to better control the amount of communication with respect to the person she is in contact with. Misunderstanding others' intentions was also less frequently observed. As a most visible change, the employer highlighted that Ms. Smith's communication style changed reflecting that she had become more considerate of others' mental state during a conversation. On the other hand, no changes in self-determination were observed. At the time of the follow-up assessment, Ms. Smith still had her job. Her employer still noticed the above-mentioned changes in her behavior. However, she added, that the changes are not as explicit as they were before. This observation was most characteristic in the field of initiating a conversation. Accordingly, the patient also indicated during the 3-months follow-up that she become uncertain about her skills, especially in complying with communication rules, but no deterioration in ToM skills was perceived by her.

### Objective Results

**Figure 2:**
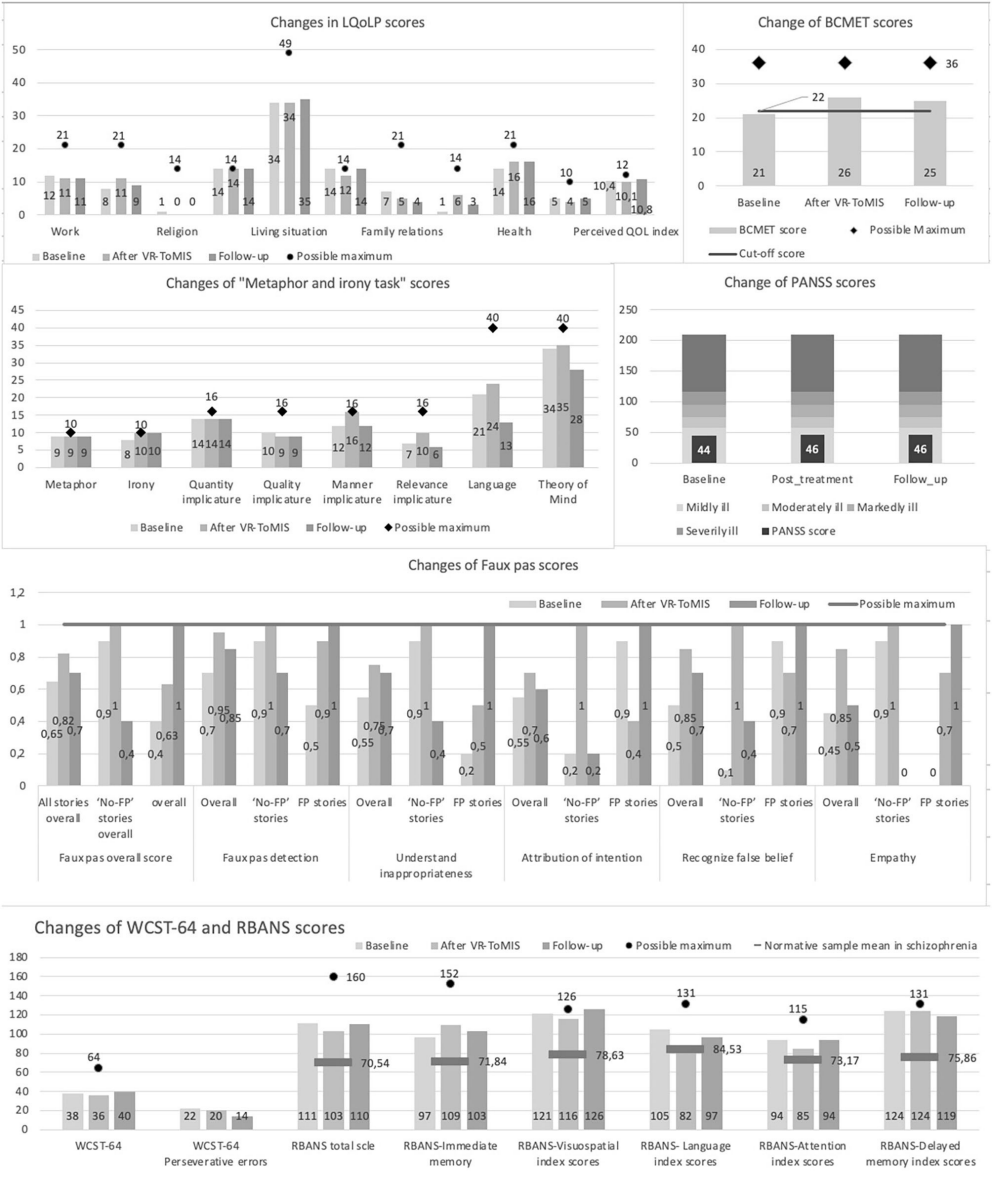
Changes is scores form pre-to post intervention and after 3-month follow-up.

Objective results were calculated compared to the baseline scores, considering the maximum scores available on the tests (Figure 2). Considering the ToM assessment, there was a 21% increase in the BCMET scores. The improvement remained at 19% at the 3-months follow-up. Cartoon total scores showed a 5% increase after the intervention but vanished at the 3-months follow-up. Almost all subscales of the Faux pas test showed improvement within the range of 11–88% after treatment. In 37% of the cases, improvement on Faux pas scores were sustainable at the follow-up assessment. Regarding pragmatic language skills, 33% improvement on manner implicatures and 44% on relevance implicatures were detected. However, in line with qualitative results 3-months after the treatment this change was no longer detectable. In contrast to subjective feedback, in the case of quantity implicatures, no changes were detected. With respect to symptomatology and neurocognition, no changes were observed after VR-ToMIS. However, our patient's scores were above the normative sample means in schizophrenia even at baseline (Loughland et al., 2007). Regarding Quality-of-Life scores, only the subjective evaluation of Social Relationships showed remarkable change, which stayed notable also at the follow-up (Figure 2). A high sense of presence and no side-effects were reported during treatment (Realism sub-score on Presence questionnaire: 83%).

## Discussion

Our case report describes a case of a chronic schizophrenic patient with good compliance. She responded well to antipsychotic medication and most of her symptoms have been alleviated. However, she still suffered from persistent social disfunctions. Lacking options to improve her complaints, she applied for our experimental VR-based intervention as soon as she heard of it.

Despite the initial difficulties, Ms. Smith enjoyed VR-ToMIS, and during the intervention she improved in a way that proved to be transferable to real life interactions and changes were even noted by her employer. Moreover, many of these improvements remained stable 3-months after the end of the intervention. With VR-ToMIS Ms. Smith learned to identify other people's mental states more accurately, which was also indicated by the BCMET and Faux pas scores. In line with literature data, suggesting the sense of presence as a core element of successful VR interventions, the mentioned experiences were associated with the subjective feeling of presence during the simulation (Diemer et al., 2015).

Consistent with the changes observed in the patient's behavior and attitude, increased scores on relevance and manner implicatures tasks were also noticed, suggesting that Ms. Smith's performance improved in complying with the main communication principles. On the other hand, implicature subtasks of Metaphor and Irony seem to be only sufficiently informative to notify the breaching the principles and not for following them. This assumption is supported by the fact that although her contribution became appropriately informative during an interaction after VR-ToMIS, this change wasn't followed by the improved scores in the quantity implicatures subtask. In contrast to our previously published pilot results, the effect of VR-ToMIS on ToM and pragmatic language skills wasn't accompanied by any change in symptomatology and neurocognition. Since the strong relationship between ToM deficits and negative symptoms is well-supported by the literature, the lack of improvement assumes a complex mechanism behind the intervention, which requires further research (with larger sample size) to be understood (Harrington et al., 2005; Sprong et al., 2007). With respect to Quality-of-life, only subjective evaluation of social relationships changed during the intervention. Surprisingly, no improvement was detected in “work” sub-scores. When we tried to explore the background of her evaluation Ms. Smith explained that, in the end, nothing changed, as she's still working at the sheltered workshop. Although low internal consistency of Work domain on the scale can also be a possible explanation, in addition to the above there were no other changes detected in Quality-of-Life domains after the intervention at the follow-up assessment (Gaite et al., 2000; Vass et al., 2020). Considering the contradiction between the objective and subjective results at this point, choosing the inappropriate measuring tool to assess the desired changes can be a probable explanation.

Beyond the results described, there are some advantages and disadvantages of using VR-ToMIS worth mentioning. It seems that VR-ToMIS may have a strong learning transfer effect, helping the patient utilize the learnt skills in everyday life, as was supported by the patient, her employer and also by our observations. This conclusion is in line with the observable trend in the development of ToM interventions, suggesting that the use of VR technology in ToM development might further the advantages of classic ToM interventions in schizophrenia (Vass et al., 2018). Furthermore, as the problem of compliance is usually a major obstacle in psychiatric care for schizophrenic patients, using a method that is interesting and enjoyable is crucial. Based on patients' feedbacks, VR-ToMIS is well-tolerated and enjoyable, which holds the promise of taking a step toward solving the problem of compliance. Additionally, this is a low budget development, which is technically based on a free downloadable software. However, although the program is well-designed and we are in contact with the company, keeping in mind their primarily entertainment purposes, the number of available virtual destinations that can be adapted to our goals is limited, which also limits our scope for expanding the intervention. In addition, VR-ToMIS requires the simultaneous use of more technical devices by the clinician, that may make use of the method challenging.

The overall experiences suggest that VR-ToMIS is well-tolerated and, despite the disadvantages, the initial results are quite promising. However, some limitations also must be mentioned. Only a pilot RCT study and a single case report suggest the feasibility of VR-ToMIS, which clearly indicates the need for further research, with a larger sample size. In addition, follow-up results on the possible long-term effects of the intervention haven't been published yet, which makes this case report a single point of reference. Finally, conflicting results of Quality-of Life scale and subjective observations on the patient's improvement highlight that the choice of appropriate assessment tools can influence the interpretation of the experiences, which we need to pay more attention to in future studies. Furthermore, in light of the observed difference between short-term effects and detectable effects during the 3-months follow-up, future studies may also consider the possibility of increasing the length of the intervention, which might be more beneficial in maintaining the improvement.

## Conclusion

The presented case suggests that VR-ToMIS may be a useful tool for rehabilitation in schizophrenia even in chronic cases where the patient exhibits severe difficulties in social functioning.

## Data Availability Statement

The datasets presented in this article are not readily available because Research data is confidential. Requests to access the datasets should be directed to Edit Vass, vass.edit@med.semmelweis-univ.hu.

## Ethics Statement

The studies involving human participants were reviewed and approved by Semmelweis University, Local Research Ethics Committee. The patients/participants provided their written informed consent to participate in this study. Written informed consent was obtained from the individual(s) for the publication of any potentially identifiable images or data included in this article.

## Author Contributions

ZF and BK collected data on the patients. EV and LS led the interventions. EV and VS wrote the paper. All authors provided critical comments and approved the final version of the manuscript.

## Conflict of Interest

The authors declare that the research was conducted in the absence of any commercial or financial relationships that could be construed as a potential conflict of interest.
